# The psychometric properties of the Career Adapt-Abilities Scale-China Short Form: factorial invariance and its longitudinal relation to boredom proneness

**DOI:** 10.3389/fpsyg.2025.1703859

**Published:** 2025-11-10

**Authors:** Yingying Hu, Gongxing Chen, Jiamiao Zhang

**Affiliations:** 1 School of Marxism, Guangxi Vocational College of Water Resources and Electric Power, Nanning, China; 2 Center for Mental Health, Guangxi Vocational College of Water Resources and Electric Power, Nanning, China

**Keywords:** career adaptability, boredom proneness, cross-lagged panel model, quality education, factorial invariance

## Abstract

Career adaptability is often measured using the Career Adapt-Abilities Scale-China Form in China. Considering the simplicity of the scale and making it easier to use, this study validated the Career Adapt-Abilities Scale-China Short Form (CAAS-CSF) for the Chinese context. Using the stratified cluster sampling method, 259 Chinese college students were selected as the research subjects for three consecutive semesters. First, gender and longitudinal factorial invariance of the CAAS-CSF were tested by multiple-group confirmatory factor analysis. Second, this study examined the concurrent validity of CAAS-CSF with regard to boredom proneness by cross-lagged panel model (CLPM). All fit indices indicated that the CAAS-CSF was factorial invariance across gender and longitudinal factorial invariance across time points. The relationship between career adaptability and boredom proneness generally suggested significant negative correlations both within and across time points. In addition, the CAAS-CSF showed good internal consistency indicators at all three time points, and a moderate to large rank-order stability coefficient was also found over 1 year in college students. The CAAS-CSF has very good psychometric properties and concurrent validity, suggesting that it is a valid measure to analyze career adaptability and apply it to the fields of education, career counseling and research.

## Introduction

Career adaptability reflects an individual's self-regulating resources to address unfamiliar, complex and unclear issues arising from developmental vocational tasks, career changes and job trauma, and is a psychosocial structure (Aminah et al., 2024; Savickas and Porfeli, 2012). Career adaptability is the core concept of Career Construction Theory (CCT) and one of the most mature theories (Soares et al., 2022). CCT explains how individuals combine their own characteristics to make career choices and give career path meaning and indicates that individuals with higher levels of career fitness are more likely to establish broader paths and opportunities, as proposed in the multicultural social context of the global economy (Savickas, 2005). Therefore, career adaptability is associated with improving individual good health and well-being.

### Career Adapt-Abilities Scale-China Short Form

To measure career adaptability, an international team from 13 countries developed the Career Adapt-Abilities Scale International Form (CAAS-IF), which consists of 24 items and four factors of concern, control, curiosity, and confidence (Savickas and Porfeli, 2012). The CAAS-IF presents good psychometric properties and has been verified in both Western and non-Western countries (Affum-Osei et al., 2021; Di Maggio et al., 2015; Dries et al., 2012; Hou et al., 2012; Lee et al., 2021; Marques et al., 2024; Mazahreh et al., 2019; Olugbade, 2016; Porfeli and Savickas, 2012; Tak, 2012). In a sample of 296 college students (age from 18 to 22 years, M = 20.07, SD = 0.83) from three universities in China, it was found that the CAAS China Form (CAAS-CF) was identical to the CAAS-IF, with a total reliability index of 0.92 and fitting indicators of RMSEA = 0.064 and SRMR = 0.057 (Hou et al., 2012). There were gender and grade-level differences in career adaptability scores, with males being higher than females, and freshmen and junior high school students being higher than sophomores (Hou et al., 2012). In order to facilitate the practical use of CAAS-IF with other tools in evaluation or organizational research, especially in large-scale surveys with time constraints, it is necessary to develop an economical and relevant alternative to CAAS-IF. Therefore, in different national backgrounds, researchers have successively developed and verified the four-factor CAAS-Short Form (CAAS-SF) based on the CAAS-IF. The earliest research team composed of Switzerland and the United States developed and tested a more concise version of CAAS-SF, consisting of 12 items, using 2,800 French and German speaking adults in Switzerland as samples, with the aim of promoting the integration of CAAS-IF into large-scale surveys in different populations and environments (Maggiori et al., 2017). Some researchers also verified the short 12 item version of CAAS-SF against the background of Türkiye, using subjects from three different age groups, and found that CAAS-SF has factor invariance across gender and age groups, and shows high enough internal consistency (Işik et al., 2018). In addition, using Portuguese college students and working people as subjects, the researchers also found that CAAS-SF is applicable to the Portuguese environment and is an effective and reliable tool for measuring occupational adaptability in both research and practice (Soares et al., 2022). These studies have consistently concluded that the CAAS-SF includes concern, control, curiosity, and confidence factors, with a total of 12 items—specifically, items 1, 3, 6, 7, 8, 9, 10, 12, 13, 14, 16, and 19 from the CAAS-CF.

Moreover, it has not been found that researchers have verified CAAS-SF (12 items) based on Chinese Mainland samples. Therefore, in this study, the Chinese version of the CAAS-SF was named The CAAS-CSF, which also includes four factors and 12 items. Although the construct validity of the different versions of the CAAS-SF was validated in some countries, using techniques from CFA or MCFA, focused on measurement invariance analyses across groups, no longitudinal invariance analyses were conducted. Therefore, this study will take college students in mainland China as samples, and on the basis of the longitudinal study, conduct cross-group and cross-time point psychometric analysis of the CAAS-CSF to test whether it is suitable for use in the context of the Chinese mainland. The evidence of structural validity can be directly provided through factor analysis. However, the concurrent validity of the CAAS-CSF also needs to be considered more. Many variables can be used to test the concurrent validity of the CAAS-CSF in measuring career adaptability, such as boredom proneness.

### Career adaptability and boredom proneness

Boredom is a widespread negative experience, which brings an inherent problem, that is, bored people want to be occupied meaningfully and do not want to participate in any currently available choices (Dadzie and Danckert, 2025). Boredom proneness refers to an individual's tendency to experience boredom (Farmer and Sundberg, 1986). People who are easily bored will feel bored more frequently and tend to be bored their whole life (Tam et al., 2021). According to the Social Cognitive Career Theory (SCCT), an individual's self-regulation ability is an important factor in gaining career achievement and satisfaction (Wang et al., 2022). Career adaptability can be achieved and improved through self-regulatory behavior (Wang and Liu, 2022), while boredom proneness may weaken the individual's self-regulatory ability. A sample of 890 college students explored the relations between leisure involvement, leisure boredom, and university life adjustment, and the result shows that leisure boredom has a negative significance in university life adjustment (Ha and Youn-Shin, 2010). A study suggested that individuals with higher levels of trait boredom experience increased difficulty in adapting, possibly because people with higher trait boredom lack self-knowledge, making it more difficult to cope with the increasing adjustment disorder symptoms (Bambrah et al., 2022). It can be seen that boredom, as an individual trait, is a tendency to experience boredom, which will reduce the individual's career adaptability. From the CCT, career adaptability reflects an individual's self-regulating resources to address unfamiliar, complex and unclear issues arising from developmental vocational tasks, career changes and job trauma, and is a psychosocial structure (Porfeli and Savickas, 2012; Savickas, 2005). The higher the level of self-control, the lower the boredom proneness (Isacescu and Danckert, 2018). Individuals with greater adaptive resources exhibit a higher level of environmental and social adaptability through self-control and self-regulation and make greater progress in reducing boredom proneness. Therefore, career adaptability may negatively predict boredom proneness through self-control or adjustment, which is a kind of psychosocial structure. In particular, we hypothesized career adaptability relates negatively to boredom proneness.

Therefore, to gather preliminary evidence of the concurrent validity of the CAAS-CSF, this study examined its relationship with a key theoretically relevant variable: boredom proneness. Using a cross-lagged panel model, we assessed the relation between career adaptability and boredom proneness.

### Current study

Although different versions of the CAAS-SF have been validated for conceptual validity in some countries using CFA or MCFA techniques (Işik et al., 2018; Maggiori et al., 2017; Soares et al., 2022), which focus on measurement invariance analyses across groups, longitudinal invariance analysis has not been conducted, nor has the concurrent validity of the CAAS-SF been verified. Additionally, no study based on Chinese mainland samples has examined the Chinese version of the CAAS-SF (CAAS-CSF) with four factors and 12 items. Therefore, it is essential to conduct psychometric analyses of the CAAS-CSF across groups and time points based on longitudinal studies in the context of the Chinese mainland, as well as to test the concurrent validity of the CAAS-CSF. This would provide researchers with an economical and relevant alternative to the CAAS-CF for conducting career force assessment and organizational research in mainland China, especially in large-scale surveys with time constraints.

Therefore, the purpose of this study was twofold. Firstly, the present study was designed to assess the psychometric properties of the CAAS-CSF. This study was conducted with Chinese college students, using the framework of multiple-group confirmatory factor analysis (MGCFA), to explore the longitudinal factorial invariance (LFI) across three time points and the gender factorial invariance (GFI) to determine whether the CAAS-CSF can be reliably and effectively detected changes or developments of career adaptability and conduct gender comparison. Secondly, the cross-lagged panel model (CLPM) was used to verify the concurrent validity of CAAS-CSF with regard to boredom proneness. Our psychometric evaluation of the CAAS-CSF adhered to the evaluative criterion checklist by Hernández et al. (2020) to ensure a valid and appropriate process. This checklist, covering planning, confirmation, and documentation phases (Hernández et al., 2020), provided a structured framework for key aspects of our study, including the tests of measurement invariance and validity, thereby ensuring methodological rigor.

## Methods

### Participants

To make the sample more representative and facilitate the data tracking of longitudinal research, this study adopts stratified cluster sampling and selects Chinese Mainland college students as the research objects. Firstly, randomly select 4 universities of different levels, including 2 undergraduate colleges and 2 vocational colleges. Secondly, randomly select 5 classes from the selected undergraduate and vocational colleges, for a total of 20 classes. Three waves of data were collected, spaced equally at 6-month intervals. The first measurement was conducted in mid-October 2021 (hereafter referred to as T1), the second measurement was conducted in mid-April 2022 (hereafter referred to as T2), and the third measurement was conducted in mid-October 2022 (hereafter referred to as T3). At T1, 802 students participated in the survey, with 537 completing all three waves and 265 attritions (a dropout rate of 33.0%). Subsequently, to ensure data quality, 13 participants who failed an attention-check item (which instructed them to select the third option) were excluded due to indications of careless responding. This yielded a final analytical sample of 259 participants, corresponding to a valid response rate of 48.2% of the longitudinal cohort. The sample was composed of 159 males (61.4%) and 100 females (38.6%), with 78% of participants from rural areas and 22% from urban areas. The age range is 18–22 years old, with an average of 18.43 and a standard deviation of 0.88.

We conducted a normality test and found that both skewness and kurtosis values for career adaptability (skewness = −0.589 ~ −0.289; kurtosis = 0.149 ~ 0.871) and boredom proneness (skewness = −0.081 ~ 0.161; kurtosis = −0.616 ~ −0.445) scores of the 259 subjects met the criteria of −1 to +1 (Tan et al., 2022), respectively. The skewness and kurtosis values for the total scores of career adaptability and boredom proneness fell within an acceptable range for normality, suggesting that the data from the selected sample do not exhibit severe deviations from a normal distribution and are suitable for subsequent parametric statistical analyses.

To assess potential bias associated with missing data, a series of *t*-tests and chi-square tests were conducted according to best practices for identifying sources of missing data (Nicholson et al., 2017). The analysis results showed that there were no significant differences in age (*t* = −1.22, *p* = 0.22), registered residence (χ^2^ = 1.01, *p* = 0.31), career adaptability (*t* = –0.37, *p* = 0.72), and boredom proneness (*t* = 0.42, *p* = 0.68) between the loss and invalid response subjects and the 259 subjects who completed all three surveys, except for gender (χ^2^ = 24.62, *df* = 1, *p* = 0.00). The loss rate for males is higher than for females. To further test the role of gender, the study examined the gender differences in career adaptability and boredom tendency at three time points. At all three time points, there were no gender differences in boredom proneness (*t* = −1.07, *p* = 0.28; *t* = −0.20, *p* = 0.85; *t* = −0.25, *p* = 0.80), however, males ‘career adaptability was higher than females' at all three time points (*t* = 3.74, *p* = 0.00; *t* = 4.76, *p* = 0.00; *t* = 4.50, *p* = 0.00). Therefore, although the loss rate for males is higher than for females, the direction of gender effects on the study variables at different time points is consistent, suggesting that the missing gender differences did not have a substantial impact on the study variables. Subsequent statistical analyses will control for gender effects, to ensure the validity of the final sample.

### Measures

#### Career Adapt-Abilities Scale-China Form

The CAAS-CF, revised by Hou, Z. and S. A. Leung, et al., has 24 items in total, including four factors: concern, control, curiosity, and Confidence (Hou et al., 2012). The scale is scored with 5 points (1 = completely inconsistent, 5 = completely consistent). The higher the score, the stronger the career adaptability. A confirmatory factor analysis (CFA) was conducted to examine the factor structure of the CAAS-CF. The results (T1: χ^2^/df = 2.53, RMSEA = 0.08, CFI = 0.91, TLI = 0.90; T2: χ^2^/df = 3.07, RMSEA = 0.09, CFI = 0.90, TLI = 0.89; T3: χ^2^/df = 3.13, RMSEA = 0.09, CFI = 0.98, TLI = 0.97) indicated a good model fit, supporting the structural validity of the scale in this study (Brown, 2006). The Cronbach's α for the CAAS-CF was 0.962 [95% CI (0.955, 0.968)] at T1, 0.970 [95% CI (0.965, 0.975)] at T2, and 0.960 [95% CI (0.952, 0.966)] at T3. The McDonald's ω for the CAAS-CF was 0.962 [95% CI (0.955, 0.968)] at T1, 0.971 [95% CI (0.965, 0.975)] at T2, and 0.960 [95% CI (0.952, 0.966)] at T3.

#### Career Adapt-Abilities Scale-China Short Form

The CAAS-CSF assesses career adapt-abilities through four factors: concern, control, curiosity, and confidence. It comprises 12 items adapted from the CAAS-CF (Hou et al., 2012), specifically items 1, 3, 4, 6, 9, 10, 11, 12, 13, 14, 16, and 19. Each item is rated on a 5-point scale, yielding a total score ranging from 12 to 60, with higher scores indicating stronger career adapt-abilities. The four factors are measured as follows: concern (items 1, 9, 13), control (items 3, 11, 19), curiosity (items 6, 10, 14), and confidence (items 4, 12, 16), with each factor consisting of three items. The factor structure of the CAAS-CSF was evaluated using confirmatory factor analysis, and the results (T1: χ^2^/df = 2.51, RMSEA = 0.08, CFI = 0.96, TLI = 0.95; T2: χ^2^/df = 3.29, RMSEA = 0.09, CFI = 0.95, TLI = 0.93; T3: χ^2^/df = 3.28, RMSEA = 0.09, CFI = 0.92, TLI = 0.94) supported good structural validity (Brown, 2006). The Cronbach's α for the CAAS-CSF was 0.930 [95% CI (0.917, 0.942)] at T1, 0.944 [95% CI (0.934, 0.954)] at T2, and 0.924 [95% CI (0.910, 0.937)] at T3. The McDonald's ω for the CAAS-CSF was 0.931 [95% CI (0.917, 0.942)] at T1, 0.945 [95% CI (0.934, 0.954)] at T2, and 0.925 [95% CI (0.910, 0.937)] at T3.

#### Boredom Proneness Scale-Short Form

The BPS-SR was used with a total of 8 items (Struk et al., 2017). The scale uses a 5-point scale (1 = completely inconsistent, 5 = completely consistent), with higher scores indicating greater boredom proneness (Struk et al., 2017). In this study, confirmatory factors of the BPS-SR were analyzed and the results (T1: χ^2^/*df* = 2.53, RMSEA = 0.08, CFI = 0.91, TLI = 0.90; T2: χ^2^/*df* = 3.07, RMSEA = 0.09, CFI = 0.90, TLI = 0.89; T3: χ^2^/*df* = 3.13, RMSEA = 0.09, CFI = 0.98, TLI = 0.97) showed that the structure validity was good (Brown, 2006). The Cronbach's α for the BPS-SR was 0.862 [95% CI (0.836, 0.886)] at T1, 0.858 [95% CI (0.830, 0.883)] at T2, and 0.868 [95% CI (0.842, 0.891)] at T3. The McDonald's ω for the BPS-SR was 0.863 [95% CI (0.836, 0.886)] at T1, 0.859 [95% CI (0.830, 0.883)] at T2, and 0.870 [95% CI (0.842, 0.891)] at T3.

### Procedure

With the informed consent of the subjects, the group test was carried out with the class as a unit, the survey was organized and unified by the psychology teacher, and ethics approval was granted. Before the test, participants were introduced to the study's purpose and provided timely guidance. No compensation was provided. The questionnaire is edited through the WJX (https://www.wjx.cn/), distributed, and collected collectively. During each measurement, it took approximately 15 min for participants to complete all questionnaires.

### Statistical methods

The data analysis employed a combination of statistical software. Preliminary data management and basic descriptive analyses were conducted using SPSS v22. All structural equation modeling (SEM) analyses, including confirmatory factor analysis (CFA), multiple-group confirmatory factor analysis (MGCFA), and the cross-lagged panel model (CLPM), were performed in Mplus 8.3 using the maximum likelihood estimation method.

The analytical procedure unfolded sequentially. First, common method bias was assessed via Harman's single-factor test. Second, item analysis (examining skewness and kurtosis) and CFA was conducted to evaluate the fundamental psychometric properties and factor structure of the measurement instruments. Following the conventional criteria suggested by Kline (2011), absolute values of skewness less than 3 and kurtosis less than 10 were used as indicative of a lack of severe violation of the univariate normality assumption. The fact that the skewness and kurtosis values meet the criterion of −1 to +1 indicates superior distribution characteristics (Tan et al., 2022). For all SEM models, model fit was evaluated using the following indices: the root mean square error of approximation (RMSEA), comparative fit index (CFI), Tucker-Lewis index (TLI), and standardized root mean square residual (SRMR). The Bayesian information criterion (BIC) was also reported for model comparison. Following conventional standards (Brown, 2006) acceptable fit was defined as RMSEA ≤ 0.08, CFI ≥ 0.90, TLI ≥ 0.90, and SRMR < 0.10, while good fit was indicated by RMSEA ≤ 0.05, CFI ≥ 0.95, TLI ≥ 0.95, and SRMR < 0.05.

Subsequently, factorial invariance across gender and time points was tested using MGCFA—a standard method for such analyses (Esnaola et al., 2019; Kline, 2011). We examined a sequence of models with increasing constraints: configural, metric, scalar, and strict invariance. Given the documented sensitivity of the likelihood ratio chi-square test to sample size, model comparisons relied on changes in practical fit indices (Cheung and Rensvold, 2002). Following established guidelines (Chen, 2007; Cheung and Rensvold, 2002), invariance was supported if the differences between nested models met the following criteria: ΔCFI ≤ 0.01, ΔTLI ≤ 0.01, and ΔRMSEA ≤ 0.015 (Esnaola et al., 2019).

After establishing the measurement model, descriptive statistics and Pearson correlation coefficients were computed for the main variables. Finally, to examine the dynamic, longitudinal relationships between the core constructs, a CLPM was specified (Burns et al., 2020; Zhou et al., 2024). This model tests the reciprocal cross-lagged effects while controlling for the stability of the constructs over time.

## Results

### Common method bias control and test

In terms of measurement procedures, this study controlled for common method bias by reverse scoring some of the questions. Prior to formal data analysis, a common method bias test was conducted using Harman's single factor test. The results revealed that the total number of factors with eigenvalues greater than 1 was 12, 10, and 11 in order in the three measurements, and the variance explained by the first factor without rotation was 35.79%, 41.09%, and 38.62%, respectively, which were all less than the 50% cut-off value (Podsakoff and Organ, 1986; Chang et al., 2010). This indicates that there was no significant common method bias across all three measurements in this study. While Harman's single-factor test is widely used, recent research has highlighted its limitations (Howard et al., 2024) including sensitivity to sample size and difficulty in effectively distinguishing true method variance from substantive trait covariance. Its effectiveness is lower than the Controlling for the Effects of an unmeasured latent methods factor (ULMC) method (Richardson et al., 2009). The ULMC method uses items directly affected by latent methods factor as its indicators, that is, based on the original trait factors, all items are used as indicators of method factors, and a bi-factor model is established (Tang and Wen, 2020). It is generally believed that if the bi-factor model differs significantly from the model containing only trait factors, then CMB is severe (Richardson et al., 2009). Using ULMC, a common method bias analysis was conducted on the data of this study again, and the difference in fitting index between the bi-factor model (χ^2^/*df* = 3,365.82/190, CFI = 0.947, TLI = 0.928, RMSEA = 0.068) and the model containing only trait factors (χ^2^/*df* = 3,365.82/190, CFI = 0.926, TLI = 0.912, RMSEA = 0.075) was very small (ΔCFI = 0.021, ΔTLI = 0.016, ΔRMSEA = −0.007), indicating that CMB is not severe. Therefore, based on Harman's single factor test and ULMC methods, it was found that there was no significant CMB in the data of this study.

### Items analysis and factor structure

The mean, standard deviation, skewness, and kurtosis are shown in Table 1. The results indicate that the skewness and kurtosis values of all items have met the criteria of −1 to +1, except for items 3, 8. This implies that the subjects scored relatively high or low on these items, which means that the difficulty of the items may be relatively low or high, or it may be due to insufficient differentiation of the items, resulting in a non-normal distribution. When the research data presents a non-normal distribution, it may lead to overestimation of the chi-square value, which in turn affects the accuracy of model fitting, as well as the reliability of parameter estimation and standard error. Maximum likelihood estimation with mean adjusted chi square is an effective method to address this challenge (Tan et al., 2022). Therefore, in the subsequent analysis, the mean adjusted chi-square maximum likelihood estimation that is robust to non-normality was used.

**Table 1:** Items analysis of the CAAS-CF and CAAS-CSF.

**Item**	**T1**				**T2**				**T3**			
	***M***	***SD***	***Sk***	***Ku***	***M***	***SD***	***Sk***	***Ku***	***M***	***SD***	***Sk***	***Ku***
**Item 1**	**3.36**	**0.91**	**−0.05**	**−0.31**	**3.47**	**0.86**	**−0.38**	**0.54**	**3.63**	**0.90**	**−0.50**	**0.23**
Item 2	3.30	0.91	−0.25	0.07	3.49	0.86	−0.42	0.39	3.59	0.90	−0.46	0.15
**Item 3**	**3.75**	**1.06**	**−0.59**	**−0.24**	**3.98**	**0.89**	**−0.97**	**1.21**	**3.92**	**0.96**	**−0.75**	**0.41**
Item 4	3.44	0.96	−0.21	−0.35	3.61	0.89	−0.45	0.07	3.66	0.94	−0.38	−0.09
Item 5	3.61	1.03	−0.41	−0.40	3.59	0.92	−0.77	0.82	3.69	0.96	−0.62	0.40
**Item 6**	**3.58**	**1.04**	**−0.52**	**−0.15**	**3.69**	**0.93**	**−0.73**	**0.55**	**3.69**	**0.93**	**−0.54**	**0.27**
**Item 7**	**3.61**	**1.06**	**−0.46**	**−0.36**	**3.73**	**0.87**	**−0.57**	**0.45**	**3.76**	**0.91**	**−0.79**	**0.88**
**Item 8**	**3.88**	**0.94**	**−0.82**	**0.63**	**3.91**	**0.83**	**−0.78**	**1.00**	**3.97**	**0.80**	**−0.85**	**1.39**
**Item 9**	**3.41**	**1.09**	**−0.50**	**−0.27**	**3.58**	**0.98**	**−0.47**	**0.04**	**3.59**	**0.93**	**−0.41**	**0.06**
**Item 10**	**3.70**	**1.01**	**−0.85**	**0.55**	**3.72**	**0.86**	**−0.55**	**0.26**	**3.85**	**0.83**	**−0.63**	**0.51**
Item 11	4.12	0.86	−1.04	1.43	4.10	0.79	−1.04	1.96	4.22	0.72	−0.60	−0.01
**Item 12**	**3.54**	**1.03**	**−0.52**	**−0.23**	**3.69**	**0.92**	**−0.74**	**0.60**	**3.71**	**0.91**	**−0.65**	**0.51**
**Item 13**	**3.49**	**1.05**	**−0.49**	**−0.11**	**3.58**	**0.97**	**−0.68**	**0.44**	**3.63**	**0.94**	**−0.62**	**0.41**
**Item 14**	**3.27**	**1.02**	**−0.22**	**−0.36**	**3.49**	**0.95**	**−0.52**	**0.24**	**3.51**	**1.00**	**−0.54**	**0.02**
Item 15	3.72	1.01	−0.63	−0.04	3.81	0.92	−0.61	0.18	3.83	0.88	−0.65	0.66
**Item 16**	**3.56**	**1.05**	**−0.50**	**−0.36**	**3.67**	**0.96**	**−0.61**	**0.21**	**3.67**	**0.92**	**−0.58**	**0.19**
Item 17	3.29	1.08	−0.29	−0.37	3.51	0.98	−0.52	0.17	3.54	1.00	−0.52	−0.12
Item 18	3.63	0.99	−0.51	−0.14	3.68	0.89	−0.72	0.81	3.77	0.83	−0.46	−0.20
**Item 19**	**3.69**	**1.03**	**−0.62**	**0.04**	**3.74**	**0.93**	**−0.76**	**0.52**	**3.80**	**0.86**	**−0.66**	**0.51**
Item 20	3.59	1.04	−0.44	−0.27	3.68	0.94	−0.63	0.21	3.76	0.92	−0.62	0.44
Item 21	3.54	1.06	−0.36	−0.41	3.61	0.94	−0.67	0.48	3.58	0.90	−0.51	0.27
Item 22	3.83	0.97	−0.76	0.32	3.76	0.95	−0.90	0.82	3.86	0.86	−0.68	0.53
Item 23	3.92	0.92	−0.66	−0.14	3.90	0.85	−0.68	0.60	4.00	0.75	−0.51	0.15
Item 24	3.45	0.96	−0.53	0.20	3.60	0.83	−0.70	0.77	3.60	0.83	−0.52	0.92
CAA	86.30	17.59	−0.46	0.15	88.60	16.76	−0.59	0.87	89.83	15.40	−0.29	0.51

M, means; SD, standard deviations; SK, Skewness; KU, Kurtosis. T1/2/3, Time 1/2/3; CAA, career adaptability. CAAS-CF contains item 1-item 24, and CAAS-CSF only contains 12 items in bold in Table 3.

Separate confirmatory factor analyses (CFAs) were conducted for the original (CAAS-CF) and short (CAAS-CSF) forms at each wave. The CAAS-CSF was supported as a more parsimonious model, demonstrating excellent absolute fit indices across all time points (T1: ΔCFI = 0.049, ΔTLI = 0.046, ΔRMSEA = −0.001; T2: ΔCFI = 0.047, ΔTLI = 0.040, ΔRMSEA = 0.005; T3: ΔCFI = 0.028, ΔTLI = 0.055, ΔRMSEA = 0.003). Considering that the CAAS-CSF is the best-fit model for observed data at each wave, therefore, CAAS-CSF was selected for subsequent factorial invariance and cross-lagged panel analysis.

### Factorial invariance

Factorial invariance refers to the consistency of the factor structure of a scale or testing tool when measuring the same latent variable in different populations or conditions, which requires that the factor loadings of the observed variable on the latent variable and the relationships between factors remain unchanged across different populations (Meredith, 1993; South et al., 2009). Configural, measurement, and structural invariance are collectively termed factorial invariance (Grugan et al., 2024; Lau and Yuen, 2015). Configural invariance is generally used as the baseline model for testing factorial invariance across groups or time points. The hierarchy of measurement invariance includes metric invariance, scalar invariance, and strict invariance error variance invariance. Structural invariance encompasses factor variance invariance, factor covariance invariance, and latent mean invariance.

The configural invariance model is established first, in which the factor loadings and thresholds are freely estimated parameters, except for those constrained for model identification. The most parsimonious model is usually the baseline model for a group, which represents the most meaningful and appropriate model for that group of data (Lau and Yuen, 2015). The process of testing measurement invariance usually begins with the testing of a baseline confirmatory factor analysis model, where items are constrained to specific factors and group or time comparisons involve basic indicators of model fitting (Dimitrov, 2010). Subsequently, more constrained models are tested:. Metric invariance is tested by constraining factor loadings to equality across groups or time points. Scalar invariance is then tested by additionally constraining the item thresholds (or intercepts) to be equal across groups or time points. The test of error variance invariance involves constraining errors or disturbances associated with each item to invariance across groups or time points (Suh et al., 2016). Similarly, for structural invariance: The test of factor variance invariance examines whether the factor variances are equivalent across groups or time points. The test of factor covariance invariance examines the factor covariances are equivalent across groups or time points. The test of latent mean invariance examines the latent variable means are equivalent across groups or time points (Lau and Yuen, 2015).

Both gender and longitudinal factorial invariance require testing a series of models with increasingly strict constraints (Dimitrov, 2010). The effects on model fit are tested for each successive set of constraints. An acceptable model fit of a less restricted model is a necessary condition for applying further model restrictions. In other words, if the model fit does not deteriorate significantly, the more constrained model is retained. This study conducted multiple-group confirmatory factor analysis to test whether the CAAS-CSF exhibits factorial invariance (including configural, measurement, and structural invariance) across gender and across time points.

#### Gender factorial invariance

Based on these criteria (ΔCFI, ΔTLI, and ΔRMSEA), and the goodness of fit indices (χ^2^, *df* , CFI, TLI, and RMSEA) for gender factorial invariance of the CAAS-CSF, we found that configural, metric, scalar, and error variance invariance were supported for the career adaptability indicators from the fitting results in Table 2.

**Table 2:** Gender factorial invariance of the CAAS-CSF.

**Model**	**χ^2^ (*df*)**	**CFI**	**TLI**	**RMSEA (90% CI)**	**Model comparison**	**ΔCFI**	**ΔTLI**	**ΔRMSEA**
M1	139.442 (96)	0.968	0.956	0.059 (0.036, 0.080)		-	-	-
**Measurement invariance**								
M2	145.091 (104)	0.969	0.961	0.055 (0.031, 0.076)	M1 vs. M2	−0.001	0.005	0.004
M3	154.766 (112)	0.968	0.962	0.054 (0.031, 0.074)	M2 vs. M3	0.001	−0.001	0.001
M4	169.489 (124)	0.966	0.964	0.053 (0.031, 0.072)	M3 vs. M4	0.002	−0.002	0.001
**Structural invariance**								
M5	158.654 (116)	0.968	0.964	0.053 (0.030, 0.073)	M3 vs. M5	0	−0.002	0.001
M6	169.151 (122)	0.965	0.962	0.055 (0.033, 0.074)	M5 vs. M6	0.003	0.002	−0.002
M7	197.973 (126)	0.946	0.944	0.066 (0.048, 0.084)	M6 vs. M7	0.019	0.018	−0.011

M1, Configural invariance; M2, Metric invariance; M3, Scalar invariance; M4, Error variance invariance; M5, Factor variance invariance; M6, Factor covariance invariance; M7, Latent mean invariance.

The structural invariance test is similar to the measurement invariance test, where the model imposes constraints on factor variance, covariance, and factor mean. Results revealed that there was no significant difference in factors of career adaptability across gender, indicating comparable individual differences in career adaptability across gender as well as equal covariance across gender. There were no significant differences in factor means across gender, indicating that the amount of career adaptability did not significantly change across gender.

In addition, comparing the means of the latent factors at the three-time point, the results indicated that the latent factor means score of the CAAS-CSF at T3 was significantly higher than T1 and T2, and at T2 was significantly higher than Time 1 (CAA T1 = 86.30, CAA T2 = 88.60, CAA T3 = 89.83; CAA T1 < CAA T2 < CAA T3; F = 15.16, *p* < 0.001).

Based on the sample data and statistical analysis collected in this study, it can be inferred that the CAAS-CSF shows configural, measurement and structural invariance across gender. Therefore, the CAAS-CSF is robust and suitable for measuring career adaptability in different groups of men and women.

#### Longitudinal factorial invariance

Table 3 presents the fitting index for longitudinal factorial invariance of the CAAS-CSF. The baseline model of the configural invariance was acceptable (CFI = 0.877, TLI = 0.867, RMSEA = 0.052). The subsequent six constraint models were constructed across time. As shown in Table 3, the three changes in fitting indicators of these models meet the cutoff criteria values, indicating that CAAS-CSF has strong longitudinal factorial invariance, and can measure changes in time for the same attribute. The results suggest that the CAAS-CSF is an appropriate tool for longitudinal mean comparisons of career adaptability.

**Table 3:** Longitudinal factorial invariance of the CAAS-CSF.

**Model**	**χ^2^ (*df*)**	**CFI**	**TLI**	**RMSEA (90% CI)**	**Model comparison**	**ΔCFI**	**ΔTLI**	**ΔRMSEA**
M1	714.984 (492)	0.958	0.946	0.042 (0.035, 0.048)		-	-	-
**Measurement invariance**								
M2	728.235 (508)	0.959	0.949	0.041 (0.034, 0.047)	M1 vs. M2	−0.001	−0.003	0.001
M3	757.351 (524)	0.956	0.947	0.041 (0.035, 0.048)	M2 vs. M3	0.003	0.002	0
M4	823.996 (547)	0.948	0.940	0.044 (0.038, 0.050)	M3 vs. M4	0.008	0.007	−0.003
**Structural invariance**								
M5	772.917 (532)	0.955	0.947	0.042 (0.035, 0.048)	M3 vs. M5	0.001	0	−0.001
M6	757.351 (524)	0.956	0.947	0.041 (0.035, 0.048)	M5 vs. M6	−0.001	0	0.001
M7	773.034 (532)	0.955	0.946	0.042 (0.035, 0.048)	M6 vs. M7	0.001	0.001	−0.001

M1, Configural invariance; M2, Metric invariance; M3, Scalar invariance; M4, Error variance invariance; M5, Factor variance invariance; M6, Factor covariance invariance; M7, Latent mean invariance.

In addition, by comparing the means of the latent factors between males and females, it was found that there were significant differences in CAAS-CSF scores between males and females, with males scoring higher than females at T1, T2, and T3 (T1: Mmale = 89.63, Mfemale = 81.01, *t* = 3.95, *p* < 0.001; T2: Mmale = 92,55, Mfemale = 82.33, *t* = 4.99, *p* < 0.001; T3: Mmale = 92.92, Mfemale = 84.92, *t* = 4.19, *p* < 0.001). The discovery of this result helps to better understand the gender differences in career adaptability and provides empirical evidence for relevant research or improvement plans on career adaptability for different gender groups. This result is consistent with a previous study (Hou et al., 2012). In Chinese cultural context, there exists a traditional notion that tends to associate men more with external pursuits and women with domestic responsibilities. This cultural inclination may lead to the perception that men exhibit greater proactivity and preparedness in career planning, potentially resulting in stronger career adaptability compared to women.

### Career adaptability and boredom proneness

#### Descriptive statistics and correlation analysis

Table 4 presents the means, standard deviations, and bivariate correlations for the main variables over time, with correlations computed as Pearson's r for continuous variables, point-biserial correlations for continuous-dichotomous variables (e.g., gender, registered residence), and phi coefficients for dichotomous-dichotomous pairs. Career adaptability (*rs* = 0.60 ~ 0.71) and boredom proneness (*rs* = 0.52 ~ 0.62) showed moderate to large rank-order stability (Cohen, 2013) over 1 year. This result suggests a stability in the psychological traits of career adaptability and boredom proneness among college students, and also reflects the stable test-retest reliability of CAAS-CSF used to test career adaptability and BPS-SR used to test boredom proneness. Career adaptability and boredom proneness were moderately (Cohen, 2013) correlated at each wave (*rs* = −0.52 ~ −0.43), in addition, across waves (*rs* = −0.52 ~ −0.34). Age was only correlated to CAA T3, and gender was correlated to CAA T1, CAA T2, and CAA T3, respectively. Therefore, in the subsequent cross-lagged panel analysis of the relations between career adaptability and boredom proneness, demographic covariates of gender and age were controlled for.

**Table 4:** Means, standard deviations, and correlations of the main variables.

**Variable**	***M***	***SD***	**1**	**2**	**3**	**4**	**5**	**6**	**7**	**8**	**9**
1. CAA T1	3.57	0.77	1								
2. CAA T2	3.69	0.72	0.65^**^	1							
3. CAA T3	3.73	0.67	0.60^**^	0.71^**^	1						
4. BP T1	2.68	0.87	−0.43^**^	−0.37^**^	0.35^**^	1					
5. BP T2	2.58	0.77	−0.36^**^	−0.52^**^	−0.45^**^	0.62^**^	1				
6. BP T3	2.66	0.81	−0.34^**^	−0.41^**^	−0.45^**^	0.52^**^	0.61^**^	1			
7. Age	18.43	0.88	0.03	0.11	0.19^**^	−0.08	−0.10	−0.08	1		
8. Gender	1.39	0.49	−0.23^**^	−0.29^**^	−0.27^**^	0.07	0.01	0.02	−0.16^**^	1	
9. RER	1.78	0.42	0.01	0.04	−0.02	−0.03	−0.11	−0.06	0.04	0.02	1

^*^p < 0.05.

^**^p < 0.01.

^***^p < 0.001.

T1/2/3, Time 1/2/3; CAA, Career Adaptability; BP, Boredom Proneness. Gender (1 = Male, 2 = Female); RER, Registered Residence (1 = Urban, 2 = Rural); For these dichotomous variables, a positive correlation indicates an association with the higher coded category (i.e., Rural Hukou or Female).

#### Cross-lagged panel model

**Figure 1:**
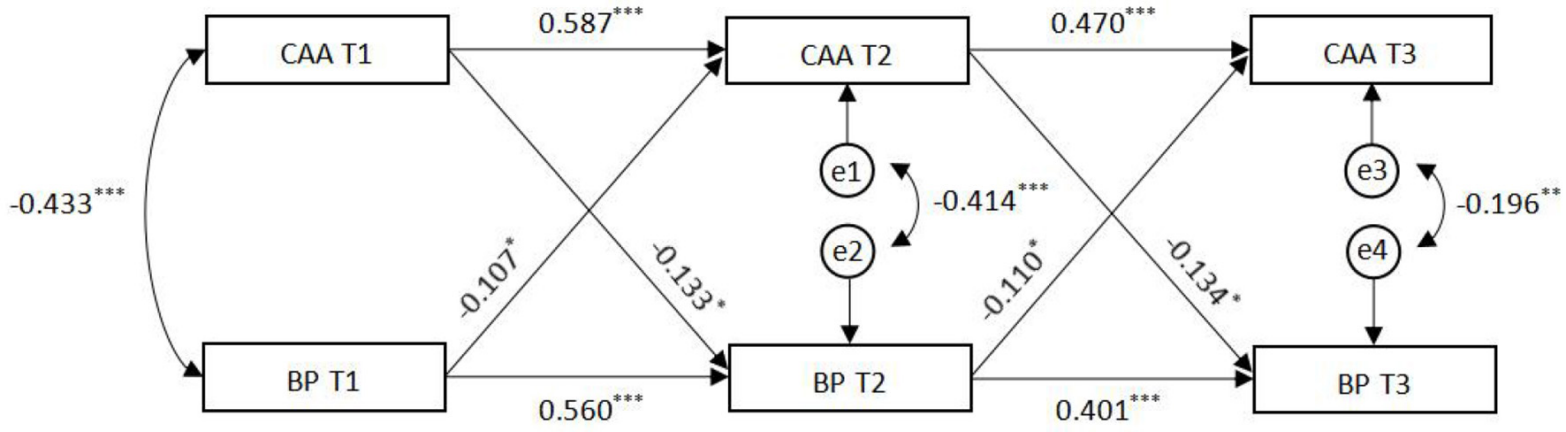
Cross-lagged relations between career adaptability and boredom proneness. Cross-lagged panel model shows longitudinal relations between career adaptability and boredom proneness. Standardized coefficients are reported. Demographic information is controlled in all paths. T1/2/3, Time 1/2/3. *p < 0.05, **p < 0.01, ***p < 0.001.

Based on the adjacent time interval of 6 months and the total time of 1 year, the longitudinal relationship between career adaptability and boredom proneness was analyzed by cross-lagged structural equation modeling, as shown in Figure 1. The cross-lagged model in this study showed a sufficient fit to the data [χ^2^ (*df* ) = 15.756(6), CFI = 0.986, TLI = 0.947, RMSEA = 0.079 (90% confidence interval = 0.032 ~ 0.128), BIC = 10091.069, SRMR = 0.059]. After controlling for gender and age, the autoregressive path coefficients of career adaptability were 0.587 and 0.470, respectively; and the autoregressive path coefficients of boredom proneness were 0.560 and 0.401 respectively. All autoregressive paths (βs = 0.401 ~ 0.587) were significant (*ps* < 0.001), suggesting that career adaptability and boredom proneness showed substantial rank-order stability. As for the cross-lagged effect, career adaptability at T1 significantly predicted boredom proneness at T2 (β = −0.133, *p* < 0.05), and career adaptability at T2 also significantly predicted boredom proneness at T3 (β = −0.134, *p* = 0.05). Meanwhile, boredom proneness at T1 significantly predicted career adaptability at T2 (β = −0.107, *p* = 0.05), and boredom proneness at T2 significantly predicted career adaptability at T3 (β = −0.110, *p* = 0.05). This shows that career adaptability and boredom proneness reciprocally predicted each other's developmental trajectories, and also suggests the CAAS-CSF has concurrent validity.

## Discussion

The CAAS-CF (24 items) is a commonly used measurement tool for career adaptability in China, but factorial invariance (FI), especially longitudinal factorial invariance (LFI), has been a neglected issue in studies. For the first time, this study examined the factorial invariance of the CAAS-CF and CAAS-CSF (12 items) in the sample of college students in the Chinese Mainland across genders and time points, contributing to the growth of career adaptability literature. At each time point, the single-dimensional structure model of the CAAS-CF and CAAS-CSF were tested, and found the CAAS-CSF had a better model fit than the CAAS-CF in college students, which is consistent with previous studies reporting that the CAAS-Short Form (CAAS-SF) has better psychometric properties than the CAAS (Işik et al., 2018; Soares et al., 2022). It further demonstrated the stability of CAAS-CSF among college students in mainland China. Meanwhile, based on the above results, the CAAS-CSF was selected for subsequent factorial invariance and cross-lagged panel analysis.

### Gender factorial invariance

This study tested the gender factorial invariance of CAAS-CSF, indicating that the CAAS-CSF shows configural, measurement and structural invariance across gender. This further suggests that the score differences between males and females may be caused by true gender differences rather than measurement biases. Therefore, the CAAS-CSF is robust and suitable for measuring career adaptability in different groups of men and women. In addition, the tracking data of this study at three time points showed that male had higher levels of career adaptability than female. Consistent with prior research (Hou et al., 2012), cultural beliefs in China continue to reflect a perspective where engaging in an unsuitable occupation is often viewed as a misstep for men, and choosing an inappropriate partner is similarly regarded as a significant error for women. Therefore, this research result also contributes to understanding the gender characteristics of college students‘ career adaptability, providing empirical evidence for researchers or educators to study or intervene in college students' career adaptability.

### Longitudinal factorial invariance

This study was the first to confirm the longitudinal factorial invariance of CAAS-CSF among college students and found that CAAS-CSF achieved strong longitudinal factorial invariance over a 12-month interval, indicating that that this tool can measure changes in time for the same attribute. Therefore, based on these results, it can be inferred that CAAS-CSF is an appropriate tool for longitudinal mean comparisons of career adaptability of Chinese college students. It is worth noting that this study tested factorial invariance of the CAAS-CSF based on three time points of college student sample data spanning 1 year. Further exploration is needed to determine whether the CAAS-CSF has longitudinal factorial invariance over longer periods of time or in other populations. Although the construct validity of the different versions of the CAAS was validated across countries, using techniques from CFA or MCFA, focused on measurement invariance analyses across groups, no longitudinal invariance analyses were conducted. Moreover, it has not been found that researchers have verified CAAS-CSF (12 items) based on Chinese Mainland samples. Therefore, this study takes College Students in Chinese Mainland as samples and conducts a psychometric analysis of the CAAS-CSF across groups and time points based on a longitudinal study. The results of this study will help more researchers choose the CAAS-CSF as a measurement tool for career adaptability, and gain a deeper understanding of the development and changes of college students' career adaptability.

An interesting phenomenon was also found in this study, in which that there was a slight but significant increase in career adaptability among college students over a 12-month interval. On the one hand, with the increase in college students' age, they pay more attention to career development and the improvement of their career adaptability, which is consistent with the result from a previous study (Rottinghaus et al., 2005). On the other hand, it may be related to the fact that the sample is Chinese college students. They are also changing from lower grades to higher grades in the 12-month interval. In this process, career guidance courses are required for every university student, which makes them pay more attention to the improvement of career adaptability.

### Internal consistency and stability coefficients

Consistent with other previous cross-sectional studies (Işik et al., 2018; Soares et al., 2022), this study found satisfactory Cronbach's alpha values for the CAAS-CSF (0.930, 0.924 and 0.944) at each time point. This result suggests a stability in the psychological traits of career adaptability among college students, and also indicated that good internal consistency of the CAAS-CSF at different time points. Furthermore, the results suggested a moderate stability coefficient (*rs* = 0.60 ~ 0.71) over 1 year, which also reflects the stable test-retest reliability of CAAS-CSF as a tool for evaluating career adaptability. Taken together, structures measured by the CAAS-CSF were confirmed to be reliable over time. Career adaptability and boredom proneness were moderately correlated at each wave (*rs* = −0.52 ~ −0.43), in addition, across waves (*rs* = −0.52 ~ −0.34). This result indicates a stable moderate correlation between career adaptability and boredom proneness among college students, and further suggests that exploring the longitudinal relationship between career adaptability and boredom proneness through this trait is very appropriate for testing the concurrent validity of CAAS-CSF.

### Cross-lagged panel model and concurrent validity

The cross-lagged panel model relations between career adaptability and boredom proneness verify that the CAAS-CSF has robust concurrent validity in this study. Both all autoregressive paths and cross-lagged effects were significant, suggesting that career adaptability and boredom proneness demonstrate substantial rank-order stability, which also implies that career adaptability and boredom proneness reciprocally predicted each other's developmental trajectories. It verifies our hypothesis that career adaptability relates negatively to boredom proneness. According to the CCT, career adaptability reflects an individual's self-regulating resources to address unfamiliar, complex and unclear issues arising from developmental vocational tasks, career changes and job trauma, and is a psychosocial structure (Savickas, 2005; Savickas and Porfeli, 2012). For this reason, when individuals face boredom tendencies, their career adaptability will promote individual self-regulation, make adjustments, give meaning, and obtain opportunities, thus reducing the level of boredom tendencies. Higher trait boredom significantly predicted increased difficulties with adapting, as those who generally experienced more frequent and intense boredom had increasing difficulty concentrating and performing other necessary tasks in daily life (Bambrah et al., 2022). Meanwhile, leisure boredom has a negative significance on university life adjustment (Ha and Youn-Shin, 2010). Because of these, the career adaptability of individuals with boredom proneness will be reduced, and this also validates the Social Cognitive Career Theory (Wang et al., 2022; Wang and Liu, 2022). Therefore, this study found a robust cross-lagged relations between career adaptability and boredom proneness, which not only validates the CAAS-CSF has concurrent validity, but also points out the direction for reducing boredom proneness by intervening in individuals' career adaptability in educational environments.

## Implications

The results of this study provide important considerations for the assessment of college students' career adaptability, as well as empirical evidence that reducing boredom tendency by intervening in college students' career adaptability in an educational environment. First, the CAAS-CSF has gender and longitudinal factorial invariance, which is suitable for use in different groups of men and women, and also suitable for longitudinal mean comparisons of career adaptability. It helps to improve the simplicity, reliability and accuracy of career adaptation assessment in career counseling. Secondly, the gender factorial invariance in the CAAS-CSF was confirmed, which demonstrated that it is appropriate and meaningful to compare the CAAS-CSF scores between different gender groups. The results across all three time points consistently indicated that male participants reported higher levels of career adaptability than their female counterparts. Given that measurement invariance of the CAAS-CSF was established, these differences likely reflect genuine disparities in the latent construct, although their precise underlying causes (e.g., social, cultural, or environmental factors) remain to be fully explored by future research. This finding provides an initial empirical basis for considering gender in the design of career adaptability interventions. Finally, we validated the concurrent validity of the CAAS-CSF and found a robust the cross-lag relations between career adaptability and boredom proneness, providing empirical evidence for reducing boredom proneness by intervening in college students‘ career adaptability in educational environments. Career adaptability has been recognized in the 21st century as a central factor in adult career development, and individuals with a higher level of career adaptability are more likely to establish broader paths and opportunities (Savickas, 2005; Savickas and Porfeli, 2012). Therefore, it is very meaningful to identify easy-to-use and effective career adaptability assessment tools (the CAAS-CSF), evaluate individuals' career adaptability, study strategies for improving career adaptability, and explore how career adaptability can reduce boredom tendencies.

## Limitations and future studies

There are still some limitations in this study. Firstly, the regional composition of the sample from western China, along with some participant attrition across the study waves, might affect the broader applicability of our findings. Future studies that include participants from more diverse regions and backgrounds would be valuable to confirm and extend these results. Secondly, at the cross-sectional level, the factorial invariance was only tested across gender. Thus, further studies should test for factorial invariance in other groups, such as the grade group. Furthermore, although this study analyzed longitudinal factorial invariance by three repeated measures over a 12-month time interval, wider time spans and more time points are required to test the stability of invariance and reveal the development of career adaptability among college students, such as 3 or 4 years of college. As a fourth limitation, it should be noted that a five-factor version of the CAAS has been developed in recent research, which incorporates a fifth factor of cooperation (Leong et al., 2023). The five-factor CAAS has not been verified in this study. Thus, this is the direction of further exploration in the study of career adaptability measurement tools. Based on item response theory and generalizability theory, five-actor CAAS and four-actor CAAS were psychometric analyzed to test their reliability and validity. Finally, to examine the concurrent validity of CAAS-CSF, we analyzed the relations between career adaptability and boredom proneness by cross-lagged panel model (CLPM). However, the criticism of CLPM is that causal paths are overestimated because they cannot distinguish between-people and within-people variation (Burns et al., 2020). The random intercept cross-lagged panel model (RI-CLPM) is proposed under this background. By including the potential intercept factor of each variable in the time wave, it can separate the stable differences between people, so that the autoregression and cross lag regression in the model are only within the scope of human influence (Yu et al., 2025). However, the use of CLPM or RI-CLPM depends on theoretical or practical needs, because both have advantages. Therefore, the researcher can consider further exploring the relations between career adaptability and boredom proneness with RI-CIPM in future research.

## Conclusion

This study indicated that the CAAS-CSF has stable moderate retest reliability and internal consistency reliability as an assessment tool of career adaptability. In addition, the CAAS-CSF was factorial invariance across gender, longitudinal factorial invariance across time points, and concurrent validity, which is suitable for use in different groups of male and female, and also suitable for longitudinal mean comparisons of career adaptability. The finding of robust cross-lagged relations between career adaptability and boredom proneness offers a solid empirical foundation for interventions designed to improve career adaptability and reduce boredom proneness among college students in educational contexts. We hope that researchers will further verify it in other countries and cross-cultural studies in the future. A shorter itemized measure usage prevents participants from fatigue, shortens response time and reduces loss probability. Therefore, the CAAS-CSF is a valid measure to analyze career adaptability and apply it to the fields of quality education, career counseling and research.

## Data Availability

The original contributions presented in the study are included in the article/supplementary material, further inquiries can be directed to the corresponding author.

## References

[B1] Affum-Osei E. Antwi C. O. Abdul-Nasiru I. Asante E. A. Aboagye M. O. Forkouh S. K. (2021). Career adapt-abilities scale in Ghana: psychometric properties and associations with individual-level ambidexterity and employees' service performance. Curr. Psychol. 40, 4647–4662. doi: 10.1007/s12144-019-00406-7

[B2] Aminah S. Hidayah N. Hanurawan F. Indreswari H. (2024). Tailoring of the career adaptabilities scale for Indonesian youth. Child Youth Serv. Rev. 166:107914. doi: 10.1016/j.childyouth.2024.107914

[B3] Bambrah V. Wyman A. Friedman E. Eastwood J. D. (2022). Examining the longitudinal associations between adjustment disorder symptoms and boredom during COVID-19. Behav. Sci. 12:311. doi: 10.3390/bs12090311 36135115 PMC9495664

[B4] Brown T. A. (2006). Confirmatory Factor Analysis for Applied Research. New York, NY: The Guilford Press.

[B5] Burns R. A. Crisp D. A. Burns R. B. (2020). Re-examining the reciprocal effects model of self-concept, self-efficacy, and academic achievement in a comparison of the cross-lagged panel and random-intercept cross-lagged panel frameworks. Br. J. Educ. Psychol. 90, 77–91. doi: 10.1111/bjep.12265 30657590

[B6] Chang S.-J. Van Witteloostuijn A. Eden L. (2010). From the editors: common method variance in international business research. J. Int. Bus. Stud. 41, 178–184. doi: 10.1057/jibs.2009.88

[B7] Chen F. F. (2007). Sensitivity of goodness of fit indexes to lack of measurement invariance. Struct. Equ. Model. 14, 464–504. doi: 10.1080/10705510701301834

[B8] Cheung G. W. Rensvold R. B. (2002). Evaluating goodness-of-fit indexes for testing measurement invariance. Struct. Equ. Model. 9, 233–255. doi: 10.1207/S15328007SEM0902_5

[B9] Cohen J. (2013). Statistical Power Analysis for the Behavioral Sciences, 2nd Edn. New York, NY: Taylor and Francis.

[B10] Dadzie V. B. Danckert J. (2025). State boredom but not boredom proneness influences judgements of agency. Pers. Individ. Dif. 236:113024. doi: 10.1016/j.paid.2024.113024

[B11] Di Maggio I. Ginevra M. C. Laura N. Ferrari L. Soresi S. (2015). Career adapt-abilities scale-italian form: psychometric proprieties with italian preadolescents. J. Vocat. Behav. 91, 46–53. doi: 10.1016/j.jvb.2015.08.001

[B12] Dimitrov D. M. (2010). Testing for factorial invariance in the context of construct validation. Meas. Eval. Couns. Dev. 43, 121–149. doi: 10.1177/0748175610373459

[B13] Dries N. Van Esbroeck R. Van Vianen A. E. M. De Cooman R. Pepermans R. (2012). Career adapt-abilities scale-belgium form: psychometric characteristics and construct validity. J. Vocat. Behav. 80, 674–679. doi: 10.1016/j.jvb.2012.01.012

[B14] Esnaola I. Benito M. Antonio-Agirre I. Axpe I. Lorenzo M. (2019). Longitudinal measurement invariance of the satisfaction with life scale in adolescence. Qual. Life Res. 28, 2831–2837. doi: 10.1007/s11136-019-02224-7 31177412

[B15] Farmer R. Sundberg N. D. (1986). Boredom proneness—the development and correlates of a new scale. J. Pers. Assess. 50, 4–17. doi: 10.1207/s15327752jpa5001_2 3723312

[B16] Grugan M. C. Olsson L. F. Vaughan R. S. Madigan D. J. Hill A. P. (2024). Factorial validity and measurement invariance of the athlete burnout questionnaire (ABQ). Psychol. Sport Exerc. 73:102638. doi: 10.1016/j.psychsport.2024.102638 38583793

[B17] Ha S.-W. Youn-Shin N. (2010). The relationships among leisure motivation, leisure involvement and the university life adjustment in leisure activities of university students. Korean J. Sport Stud. 49, 331–342.

[B18] Hernández A. Hidalgo M. D. Hambleton R. K. Gómez-Benito J. (2020). International test commission guidelines for test adaptation: a criterion checklist. Psicothema 32, 390–398. doi: 10.7334/psicothema2019.306 32711675

[B19] Hou Z.-J. Leung S. A. Li X. Li X. Xu H. (2012). Career adapt-abilities scale—China form: construction and initial validation. J. Vocat. Behav. 80, 686–691. doi: 10.1016/j.jvb.2012.01.006

[B20] Howard M. C. Boudreaux M. Oglesby M. (2024). Can Harman's single-factor test reliably distinguish between research designs? Not in published management studies. Eur. J. Work Organ. Psychol. 33, 790–804. doi: 10.1080/1359432X.2024.2393462

[B21] Isacescu J. Danckert J. (2018). Exploring the relationship between boredom proneness and self-control in traumatic brain injury (TBI). Exp. Brain Res. 236, 2493–2505. doi: 10.1007/s00221-016-4674-9 27215775

[B22] Işik E. Yegin F. Koyuncu S. Eser A. Çömlekciler F. Yildirim K. (2018). Validation of the career adapt-abilities scale–short form across different age groups in the Turkish context. Int. J. Educ. Vocat. Guid. 18, 297–314. doi: 10.1007/s10775-018-9362-9

[B23] Kline R. B. (2011). Principles and Practice of Structural Equation Modeling, 3rd Edn. New York, NY: Guilford Press.

[B24] Lau W. W. F. Yuen A. H. K. (2015). Factorial invariance across gender of a perceived ICT literacy scale. Learn. Individ. Differ. 41, 79–85. doi: 10.1016/j.lindif.2015.06.001

[B25] Lee I. H. Sovet L. Banda K. Kang D.-K. Park J.-H. (2021). Factor structure and factorial invariance of the career adapt-abilities scale across Japanese and South Korean college students. Int. J. Educ. Vocat. Guid. 21, 241–262. doi: 10.1007/s10775-020-09440-5

[B26] Leong F. T. L. Gardner D. M. Nye C. D. Prasad J. J. (2023). The five-factor career adapt-abilities scale's predictive and incremental validity with work-related and life outcomes. J. Career Dev. 50, 860–882. doi: 10.1177/08948453221138301

[B27] Maggiori C. Rossier J. Savickas M. L. (2017). Career adapt-abilities scale–short form (CAAS-SF): construction and validation. J. Career Assess. 25, 312–325. doi: 10.1177/1069072714565856

[B28] Marques C. Oliveira Í. M. Vautero J. Silva A. D. (2024). Career adapt-abilities scale: psychometric properties in a Lebanese sample. Int. J. Educ. Vocat. Guid. 24, 479–499. doi: 10.1007/s10775-022-09565-9

[B29] Mazahreh L. G. Stoltz K. B. Wolff L. A. (2019). Validation of the career adapt-abilities scale in the Hashemite Kingdom of Jordan. Meas. Eval. Couns. Dev. 52, 108–118. doi: 10.1080/07481756.2017.1358058

[B30] Meredith W. (1993). Measurement invariance, factor analysis and factorial invariance. Psychometrika 58, 525–543. doi: 10.1007/BF02294825

[B31] Nicholson J. S. Deboeck P. R. Howard W. (2017). Attrition in developmental psychology: a review of modern missing data reporting and practices. Int. J. Behav. Dev. 41, 143–153. doi: 10.1177/0165025415618275

[B32] Olugbade O. A. (2016). The career adapt-abilities scale-Nigeria form: psychometric properties and construct validity. J. Vocat. Behav. 95–96, 111–114. doi: 10.1016/j.jvb.2016.08.006

[B33] Podsakoff P. M. Organ D. W. (1986). Self-reports in organizational research: problems and prospects. J. Manag. 12, 531–544. doi: 10.1177/014920638601200408

[B34] Porfeli E. J. Savickas M. L. (2012). Career adapt-abilities scale-USA form: psychometric properties and relation to vocational identity. J. Vocat. Behav. 80, 748–753. doi: 10.1016/j.jvb.2012.01.009

[B35] Richardson H. A. Simmering M. J. Sturman M. C. (2009). A tale of three perspectives: examining post hoc statistical techniques for detection and correction of common method variance. Organ. Res. Methods 12, 762–800. doi: 10.1177/1094428109332834

[B36] Rottinghaus P. J. Day S. X. Borgen F. H. (2005). The career futures inventory: a measure of career-related adaptability and optimism. J. Career Assess. 13, 3–24. doi: 10.1177/1069072704270271

[B37] Savickas M. L. (2005). The Theory and Practice of Career Construction. Hoboken, NJ: John Wiley & Sons.

[B38] Savickas M. L. Porfeli E. J. (2012). Career adapt-abilities scale: construction, reliability, and measurement equivalence across 13 countries. J. Vocat. Behav. 80, 661–673. doi: 10.1016/j.jvb.2012.01.011

[B39] Soares J. Taveira M. D. C. Barroso P. Silva A. D. (2022). Career adapt-abilities scale–short form: validation among Portuguese university students and workers. J. Career Assess. 31, 571–587. doi: 10.1177/10690727221129281

[B40] South S. C. Krueger R. F. Iacono W. G. (2009). Factorial invariance of the Dyadic Adjustment Scale across gender. Psychol. Assess. 21, 622–628. doi: 10.1037/a0017572 19947795 PMC2788300

[B41] Struk A. A. Carriere J. S. A. Cheyne J. A. Danckert J. (2017). A short boredom proneness scale: development and psychometric properties. Assessment 24, 346–359. doi: 10.1177/1073191115609996 26467085

[B42] Suh H. Rice K. G. Choi C.-C. Van Nuenen M. Zhang Y. Morero Y. . (2016). Measuring acculturative stress with the SAFE: evidence for longitudinal measurement invariance and associations with life satisfaction. Pers. Individ. Dif. 89, 217–222. doi: 10.1016/j.paid.2015.10.002

[B43] Tak J. (2012). Career adapt-abilities scale — Korea form: psychometric properties and construct validity. J. Vocat. Behav. 80, 712–715. doi: 10.1016/j.jvb.2012.01.008

[B44] Tam K. Y. Y. Van Tilburg W. A. P. Chan C. S. (2021). What is boredom proneness? A comparison of three characterizations. J. Pers. 89, 831–846. doi: 10.1111/jopy.12618 33484603

[B45] Tan Q. Zou J. Kong F. (2022). Longitudinal and gender measurement invariance of the gratitude questionnaire in Chinese adolescents. Psychol. Rep. 125, 3209–3223. doi: 10.1177/00332941211036015 34338074

[B46] Tang D. Wen Z. (2020). Statistical approaches for testing common method bias: problems and suggestions. J. Psychol. Sci. 43, 215–223. Chinese.

[B47] Wang D. Liu X. (2022). The effects of cognitive information processing and social cognitive career group counseling on high school students' career adaptability. Front. Psychol. 13:990332. doi: 10.3389/fpsyg.2022.990332 36118429 PMC9480515

[B48] Wang D. Liu X. Deng H. (2022). The perspectives of social cognitive career theory approach in current times. Front. Psychol. 13:1023994. doi: 10.3389/fpsyg.2022.1023994 36533045 PMC9749854

[B49] Yu J.-M. Liu R.-D. Ding Y. Zhen R. (2025). Academic engagement lowered children's fixed mindset in mathematics: a random intercepts cross-lagged panel model with five waves. Learn. Individ. Differ. 117:102595. doi: 10.1016/j.lindif.2024.102595

[B50] Zhou S. Guan Q. Feng K. Leng M. Ma X. Zhou W. (2024). Longitudinal relationships between physical activity, body appreciation, and proactive coping in college students: a cross-lagged panel model. Body Image 51:101814. doi: 10.1016/j.bodyim.2024.101814 39531754

